# On-Site Biolayer Interferometry-Based Biosensing of Carbamazepine in Whole Blood of Epileptic Patients

**DOI:** 10.3390/bios11120516

**Published:** 2021-12-15

**Authors:** Sumin Bian, Ying Tao, Zhoule Zhu, Peixi Zhu, Qiqin Wang, Hemmings Wu, Mohamad Sawan

**Affiliations:** 1 CenBRAIN Labs, School of Engineering, Westlake University, Hangzhou 310024, China; 2 College of Pharmaceutical Sciences, Zhejiang University of Technology, Hangzhou 310014, China; taoying@westlake.edu.cn (Y.T.); zhupeixi@zjut.edu.cn (P.Z.); 3 Department of Neurosurgery, The Second Affiliated Hospital of Zhejiang University School of Medicine, Hangzhou 310009, China; zhuqianle1231@zju.edu.cn (Z.Z.); hemmings@zju.edu.cn (H.W.); 4 Institute of Pharmaceutical Analysis, College of Pharmacy, Jinan University, Guangzhou 510632, China; qiqinxtu@163.com

**Keywords:** epilepsy, biosensor, fiber-optic biolayer interferometry, carbamazepine, whole blood, signal enhancement

## Abstract

On-site monitoring of carbamazepine (CBZ) that allows rapid, sensitive, automatic, and high-throughput detection directly from whole blood is of urgent demand in current clinical practice for precision medicine. Herein, we developed two types (being indirect vs. direct) of fiber-optic biolayer interferometry (FO-BLI) biosensors for on-site CBZ monitoring. The indirect FO-BLI biosensor preincubated samples with monoclonal antibodies towards CBZ (MA-CBZ), and the mixture competes with immobilized CBZ to bind towards MA-CBZ. The direct FO-BLI biosensor used sample CBZ and CBZ-horseradish peroxidase (CBZ-HRP) conjugate to directly compete for binding with immobilized MA-CBZ, followed by a metal precipitate 3,3′-diaminobenzidine to amplify the signals. Indirect FO-BLI detected CBZ within its therapeutic range and was regenerated up to 12 times with negligible baseline drift, but reported results in 25 min. However, Direct FO-BLI achieved CBZ detection in approximately 7.5 min, down to as low as 10 ng/mL, with good accuracy, specificity and negligible matric interference using a high-salt buffer. Validation of Direct FO-BLI using six paired sera and whole blood from epileptic patients showed excellent agreement with ultra-performance liquid chromatography. Being automated and able to achieve high throughput, Direct FO-BLI proved itself to be more effective for integration into the clinic by delivering CBZ values from whole blood within minutes.

## 1. Introduction

Epilepsy is a neurological disorder characterized by an enduring predisposition to spontaneous epileptic seizures, and affects over 70 million people worldwide [1,2]. The high disability and fatality rates in epilepsy constitute heavy global burdens, particularly in low- and middle-income countries [3]. To date, antiepileptic drugs remain the first-line treatment, which a majority of epileptic patients needing to take them daily, despite being effective in only 60–70% of individuals [4]. Among the available antiepileptic drugs, carbamazepine (CBZ) is the most commonly used drug in clinical practice [5]. CBZ has a narrow therapeutic index and is a highly variable drug. Therapeutic drug monitoring (TDM) is considered a powerful tool for maximizing efficacy [6,7]. Particularly, TDM helps to investigate the effectiveness, pharmacokinetics, tolerability, dosage, and the possibility of withdrawing during CBZ treatment [1]. In clinical care, the therapeutic reference range for CBZ is 4–12 µg/mL in plasma and 2–12 µg/mL in serum [8].

Substantial efforts have been made to develop novel technologies to sensitively detect CBZ in multiple biological media, as reviewed recently [9,10,11]. A majority of the CBZ bioassays are based on enzyme-linked immunosorbent assay (ELISA), high-performance liquid chromatography, and nanomaterial-based electrodes. The former two are benchmark methods routinely conducted in hospitals because of their reliable analytical performance, even when applied to tough samples such as dried blood spots [12]. The latter can be developed into miniaturized devices for selectively detecting CBZ via a molecular imprinted polymer, an artificial antibody, as the sensing element [13]. The most appropriate tool for monitoring CBZ is an in vivo microchip-based wireless device that obtains drug levels inside the body in real time [14]. However, this requires substantial efforts to achieve. Till this develops, clinical practice urgently needs on-site TDM that goes beyond benchmark assays to provide quick, sensitive CBZ results in easy operation for individualized treatment [15]. To achieve on-site TDM, a portable nano-plasmonic electrical field-enhanced resonating device and an immunochromatographic strip were built, but both failed to provide quantitative information on CBZ levels [16,17]. A suspension array fluorescence immunoassay that was developed to detect CBZ in wastewater has the potential to be applied for TDM of CBZ in clinical settings [18]. Surface plasmon resonance biosensors have long proven their capacity in detecting various small molecules [19,20]. Another optical technique, biolayer interferometry, has gained increasing attention recently for drug analysis, in addition to its functions for kinetic study, drug discovery and vaccines research [21,22,23].

**Scheme 1:**
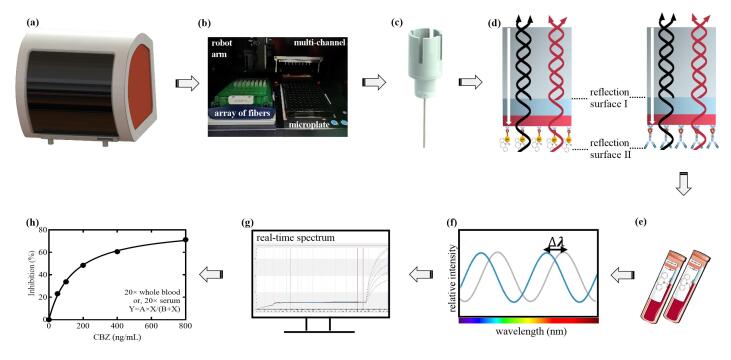
Illustration of the fiber-optic-based biosensor—experimental setup and sensing-on-fiber working principle: (**a**) automated system, (**b**) multichannel operation, (**c**) optical fibers, (**d**) sensing-on-fiber tip, (**e**) targets in blood, (**f**) internal spectrum, (**g**) data acquisition, and (**h**) data processing.

Fiber-optic biolayer interferometry (FO-BLI) is an automated system and uses a robot arm to control multichannel for automated operation, as shown in Scheme 1. Integration of multiple channels with microplates containing up to hundred samples enables multiplexed detection simultaneously and with high throughout. Fibers are made of glass with openings to connect to the robot arm and tips to interact with bioliquids. The three-dimensional fiber structure is shown in another work by our team, proving the capacity of FO-BLI for different applications [24]. The ends of the fiber tips contain two reflection surfaces (I and II)—the former serves as internal reference and the latter serves as an active probe to capture targets. When vertical light is applied, the white light reflected from interfaces I and II forms an interference pattern. Fiber tips can be coated with specific sensing elements to capture targets in blood. Molecules bound cause a change in thickness in surface II and a further change in wavelength shifts in the interference pattern. During test, shifts are acquired automatically in real time and further processed to generate specific binding curves to interpolate the unknown.

**Figure 1:**
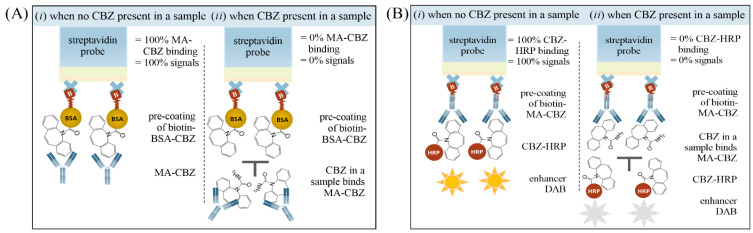
The principle of on-site CBZ monitoring for the (**A**) indirect FO-BLI biosensor via indirect competitive binding and (**B**) direct FO-BLI biosensor via direct competitive binding.

This study investigates the potential of applying the FO-BLI technique for rapid, sensitive, and automated detection of small molecule CBZ in whole blood. One concern is that CBZ has a low mass and therefore fails to generate a large BLI signal above the sensor resolution threshold of approximately 0.01 nm. To address this concern, this study used two formats (indirect versus direct) of competitive binding to obtain sufficient signals for sensitive detection. The indirect FO-BLI biosensor includes sample preincubation with monoclonal antibodies towards CBZ (MA-CBZ), and immobilized biotinylated bovine serum albumin-CBZ conjugate (biotin-BSA-CBZ) competes for binding with MA-CBZ (Figure 1A). The direct FO-BLI biosensor allows the CBZ in the samples and CBZ-BSA-horseradish peroxidase conjugate (CBZ-BSA-HRP) to directly compete for binding with immobilized biotin-MA-CBZ. Residual CBZ-BSA-HRP reacts with a metal precipitate 3,3′-diaminobenzidine (DAB) to amplify the signals in an effort to achieve higher sensitivity (Figure 1B). After establishing both biosensors, strategies to control matric interreference (when sensors are used for single or several times) or biofouling (when sensors are used for continuous measurements) from blood on the sensor surface were also studied [25,26]. Comparison between the two biosensors included their performance in terms of sensitivity, selectivity, accuracy, time to result, and regeneration ability. The better performer was further evaluated ex vivo regarding its clinical utility using several paired sera and whole blood from CBZ-treated epileptic patients.

## 2. Materials and Methods

### 2.1. Materials

CBZ, valproate sodium, phenytoin sodium, primidone, Tween-20 and bovine serum albumin (BSA) were purchased from Sigma-Aldrich (Shanghai, China). CBZ antibody clone CA1 was obtained from Bio-Rad Laboratories (Shanghai, China). CBZ bovine serum albumin conjugate was purchased from Unibiotest Co., Ltd. (Wuhan, China). Zeba^TM^ spin desalting columns (7 K MWCO, 0.5 mL), 3,3′- diaminobenzidine (DAB) enhanced liquid substrate system tetrahydrochloride, and SuperBlock™ (PBS) blocking buffer were obtained from Thermo Scientific (Shanghai, China). The biotinylating kit was obtained from Genemore (Suzhou, China). The horseradish peroxidase (HRP) Conjugation Kit-Lightning-Link^®^ (ab102890) was from Abcam (Shanghai, China). Single donor human serum off the clot and human whole blood was obtained from Innovative Research (Novi, MI, USA). The 96-well polystyrene black microplates were obtained from Greiner Bio-One GmbH (Shanghai, China). The Octet^®^ K2 2-channel system and streptavidin sensors were purchased from Sartorius Stedim Biotech GmbH (Gottingen, Germany). Phosphate-buffered saline (PBS, 10 mM, pH 7.4), PBS buffer containing 0.02% (*v*/*v*) Tween-20 and 0.1% BSA (referred to as sample diluent (SD) buffer), and SD buffer containing 274 mM NaCl (referred to as high-salt SD buffer) were used in this study.

### 2.2. Isothermal Titration Calorimetry (ITC)

ITC was conducted using a MicroCal PEAQ-ITC microcalorimeter (Malvern Panalyical Limited, Malvern, Worcs, UK). The low-ionic-strength PBS buffer was used to disperse both CBZ and monoclonal antibody towards CBZ (MA-CBZ) to avoid background interference from the heat caused by the mixing of buffers. Prior to each measurement, all the solutions were degassed to avoid bubbles. Then, a 6.67 µM antibody was loaded in the ITC cell (0.3 mL) at 25 °C. CBZ (66.7 µM) at 25 °C was titrated (2 μL each time) into the cell through a syringe, except for the first injection (0.4 μL). All thermodynamic data (enthalpy (ΔH) and dissociation constant (Kd) were auto-analyzed with the MicroCal PEAQ-ITC Analysis Software using nonlinear least squares curve fitting. The profiles were reproduced by fitting the titration curves to a nonlinear regression (curve fit) model using GraphPad Prism.

### 2.3. Bioconjugation with Biotin and Horseradish Peroxidase

CBZ-BSA and MA-CBZ were biotinylated according to the protocol of a commercial biotinylating kit and purified by removing free biotin via short centrifugation at 1500× *g* for 2 min using desalting columns. CBZ-BSA was conjugated with HRP according to the manufacturer’s protocol to obtain a stock concentration of 1.0 mg/mL in PBS. All bioconjugates were stored at 4 °C.

### 2.4. Strategies to Reduce FO-BLI Biosensor Biofouling Caused by Blood on Sensor Surface

Strategies applied to reduce biofouling on the sensor surface caused by blood included the followings: the dilution of serum and blood, use of high-salt SD buffer (SD buffer containing 270 mM NaCl), use of high-BSA SD buffer (SD buffer containing 1.0% BSA), and addition of Superblock buffer after surface functionalization. The objectives were to remove non-specifically adsorbed biomolecules from the sensor surface and to block excess binding sites. Antifouling performance of each strategy was assessed by comparing the wavelength shifts of two quality control samples with that obtained in pure SD buffer. The two controls have initial concentrations of 0 and 0.5 µg/mL for the indirect FO-BLI biosensor, and of 0 and 100 ng/mL for the direct FO-BLI biosensor.

### 2.5. Establishing an Indirect FO-BLI Biosensor for CBZ Detection in Buffer, Serum, and Whole Blood

Biotinylated BSA-CBZ coated onto streptavidin sensors was applied to detect CBZ via indirect competitive interaction. In the bioassay, free CBZ in a sample competed with immobilized biotin-CBZ-BSA for specific binding to the MA-CBZ, which was pre-added to the sample solution. Therefore, this bioassay was defined as an indirect FO-BLI biosensor. The higher the amount of CBZ present, the less MA-CBZ bound to the sensor, the fewer binding shifts were obtained. In particular, a loading shift of 1.5–2.0 nm was proposed when immobilizing the biotin-BSA-CBZ in order to have sufficient binding towards the target. CBZ was detected in three sample matrices: (i) in SD buffer with CBZ spiked from 0 to 4 µg/mL: 0—0.1—0.5—1.0—2.0—3.0—4.0 µg/mL, (ii) in 5× human serum and (iii) in 5× whole blood spiked with CBZ into a high-salt SD buffer within the detection range of 0 to 4.0 µg/mL. Spiked samples were mixed with a certain amount of MA-CBZ dissolved in the corresponding buffer in equal volumes for 20 min at 25 °C prior to analysis. The washing step was conducted between the steps to remove any unbound molecules. The generated signals were proportional to the amount of MA-CBZ residual in the sample mixture and inversely proportional to the amount of CBZ in the sample. The shifts of CBZ at 0 ng/mL were set as the reference, and the inhibition per point per sample was calculated as shown in Equation (1).

(1) $$Inhibition=\left(1-\left(\frac{shift\;value\;of\;sample}{shift\;value\;of\;negative\;control}\right)\right)\times 100\;$$

### 2.6. Establishing a Direct FO-BLI Biosensor for CBZ Detection in Buffer, Serum and Whole Blood

Biotinylated MA-CBZ was coated to allow direct competitive binding between free CBZ in a sample and pre-added CBZ-HRP conjugate, therefore being defined as a direct FO-BLI biosensor. Similarly, the lower the amount of CBZ present, the higher the amount of CBZ-HRP captured, and the higher the signals observed. Signals were amplified by submerging the sensors into the DAB solution, a metal precipitating substrate. The interaction of DAB with HRP generated precipitation on the fiber tip within seconds and induced changes in wave-length shifts. An appropriate concentration of CBZ-BSA-HRP was selected by comparing the shifts obtained from the series concentrations at 20—10—2—0.2—0.1 µg/mL. Optimal shifts for the highest target concentration were expected to reach approximately 15 nm. A 200× dilution was fixed for DAB by adding 5 µL DAB to 995 µL of substate buffer. In this bioassay, the loading shifts for biotin-MA-CBZ were aligned to be ap-proximately 1.5 nm for target capture. Similarly, CBZ was detected in three biological matrices: (i) in high-salt SD buffer with CBZ spiked from 0 to 400 ng/mL: 0—10—50—100—200—400 ng/mL, (ii) in 20× human serum, and (iii) in 20× whole blood spiked 0—400 ng/mL CBZ into high-salt SD buffer. Equal volume of CBZ-BSA-HRP was added to samples prior to analysis. Washing step was included in between. The inhibition per sample was determined followed Equation (1).

### 2.7. The Sensitivity, Specificity and Accuracy of the Two FO-BLI CBZ Biosensors

The limit of detection (LoD) was defined as the lowest CBZ concentration of the standard curve that could be detected but not reliably quantified with a coefficient of variation (CV) > 20%. The lower limit of quantification (LLoQ) was defined as the lowest CBZ concentration of the standard curve reliably measured with a CV ≤ 20%. The specificity was evaluated using three other anti-epileptic drugs, valproate sodium, phenytoin sodium, and primidone, at therapeutic concentrations of 100, 20, and 12 µg/mL, respectively. The accuracy was determined using a quality control sample of CBZ (6 µg/mL) to calculate its back recovery.

### 2.8. Regeneration of the Functionalized Optical Fibers

To evaluate whether the optical fibers could be regenerated and reused multiple times, a batch of regeneration buffers was used to evaluate the recovery of the wavelength shifts obtained at 0 µg/mL of CBZ. Regeneration solutions included 10 mM glycine (pH 2.0, 1.0, and 0.5, respectively), HCl (pH 0.5), 500 mM phosphoric acid, 5 M NaOH, and 0.01% sodium dodecyl sulfate. Regeneration was conducted via the ‘basic kinetic experiment’ model and three cycles of 10 s incubation in regeneration solution and 10 s in neutralization solution (high-salt SD buffer). Detection was conducted using the high-salt SD buffer to wash in between and at a shaking speed of 400 rpm at 30 °C. In the indirect FO-BLI, fibers were pre-loaded with biotin-BSA-CBZ to attain signals between 1.5 and 2.0 nm and further associated with the MA-CBZ at 10 µg/mL for 5 min to achieve signals at approximately 3.5 nm (referred to as Cycle 1). Afterwards, regeneration and neutralization were applied up to 12 times to measure the shifts of MA-CBZ at 10 µg/mL (referred to as Cycle 2, Cycle 3 to Cycle 12) and compared with that obtained in Cycle 1 for assessing the recovery. In the direct FO-BLI, fibers were pre-functionalized with biotin-MA-CBZ to attain a signal threshold of approximately 1.60 nm. Then, fibers were loaded with the CBZ-BSA-HRP conjugate at 1.0 µg/mL for 5 min and associated with DAB enhancer for 2 min, resulting in a sample shift at approximately 20 nm (referred to as Cycle 1). Afterwards, regeneration and neutralization were applied up to 12 times to measure the shifts of the same sample (consisting of 5 min loading of CBZ-BSA-HRP, 30 s washing, and 2 min DAB reaction) and compared with the shift obtained in Cycle 1.

### 2.9. Clinical Validation and Benchmarked against Ultra-Performance Liquid Chromatography

To evaluate the clinical utility of the FO-BLI biosensor, seven adult patients with epilepsy, who were prescribed regular CBZ drugs, were enrolled, as approved by the Institutional Review Boards of both Westlake University (20210608BSM001) and Second Affiliated Hospital of Zhejiang University School of Medicine (Research 2021-0778). Informed consent was obtained from all the participants. Sera and whole blood were collected between 4 and 5 h following the administration of conventional release tablets per individual and de-identified afterwards. Whole blood was collected by venipuncture in CBZ-treated epileptic patients using BD Vacutainer^®^ Lithium Heparin tubes. Sera were collected into the BD Vacutainer^®^ SSTTM II Advance Tubes simultaneously and centrifuged at 2000 rpm for 10 min at 25 °C to collect the supernatants. Each paired sample was measured four times in different runs by the selected FO-BLI biosensor. The inter-run variation was determined by dividing the standard deviation by the mean of the four separate measurements. As the biosensor was established to detect CBZ in whole blood, the relationship between whole blood and serum concentrations in adult epileptic patients was assessed by calculating the whole blood/serum ratio per paired. Measurements of the samples by the ultra-performance liquid chromatography (UPLC) followed the protocol as illustrated in the Supplementary Figure S1.

### 2.10. Statistics

To reduce the amount of data for profiling the flowchart, the data per five readout points were assigned into and the means were calculated via the “aggregate” function in SPSS Statistics V.26 (IBM, Armonk, NY, USA). To quantify the correlation and agreement of CBZ measurements determined by the FO-BLI biosensor and the UPLC, GraphPad Prism 9.02 (GraphPad Software, San Diego, CA, USA) was used to calculate the Pearson r correlation coefficient and SPSS Statistics V.26 (IBM, Armonk, NY, USA) was used to calculate the intra-class correlation coefficient (ICC). The ICC calculation adopted the “two-way mixed single measure test (absolute agreement)”. Differences between the CBZ measurements obtained from the two methods were analyzed using the two-tailed t-test in GraphPad Prism. Inhibition curves of both FO-BLI biosensors were determined via “dose–response-inhibitor: log (inhibitor) vs. normalized response”. The level of statistical significance was set at *p* < 0.05.

## 3. Results

### 3.1. Evaluation of Affinity Binding by ITC

**Figure 2:**
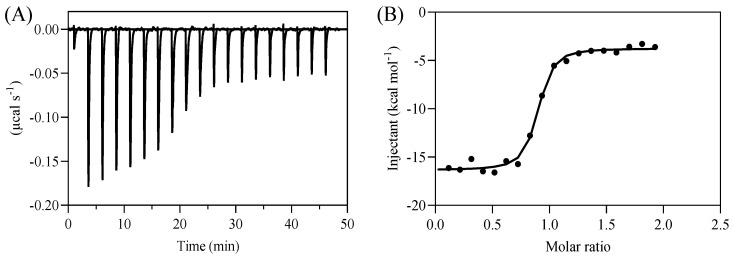
ITC analysis of the affinity between CBZ and its antibody: (**A**) The binding isotherm from the integrated thermogram fit using the one-site binding mode, (**B**) The determined molar ratio between CBZ and its antibody.

The Kd of CBZ binding was determined using ITC. Titrating CBZ to MA-CBZ released heat, and the integrated heat was fitted to a one-site binding model after subtracting the back-ground values (Figure 2A). The Kd was determined to be 30.8 ± 8.47 μM, revealing a tight and close to 1:1 (N(sites): 0.857 ± 0.009) binding between CBZ and its antibody (Figure 2B).

### 3.2. Indirect FO-BLI Biosensor for Detecting CBZ in Buffer, Serum, and Whole Blood

**Figure 3:**
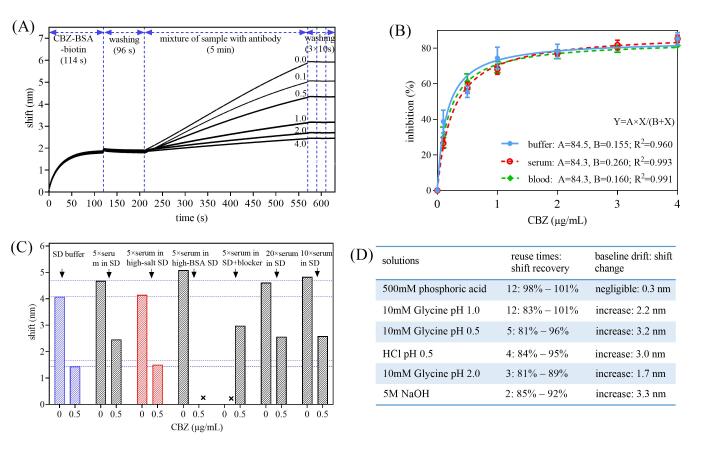
Indirect FO-BLI biosensor for CBZ detection in buffer, serum, and whole blood. (**A**) General detection flowchart in buffer; (**B**) non-linear dose-concentration binding curves in buffer, 5× serum and 5× whole blood. Error bars represent standard deviations (*n* = 3 for buffer, *n* = 2 for serum and blood); (**C**) various strategies to control non-specific binding from serum components. The two upper dashed lines represent the shift of CBZ at 0 µg/mL in pure SD buffer and its 115% signal criteria line, and the two bottom dashed lines represent the shift of 0.5 µg/mL CBZ in pure SD buffer and its 85% signal criteria line; (**D**) summary of reuse times and baseline drifts under five solutions that could regenerate the sensors.

In the indirect FO-BLI biosensor, residual binding of unbound MA-CBZ in the sample mixture resulted in a wavelength shift that was negatively correlated with the concentration of CBZ in the sample. The large mass of antibodies led to maximal shifts at 4 nm, which was sufficient for establishing a sensitive CBZ biosensor without a signal enhancer. The flowchart in Figure 3A reveals the entire detection process for the indirect FO-BLI. In particular, the detection of a sample mixture required 5 min. The coating of biotin-BSA-CBZ for capturing (taking 114 s) and washing to remove unbound biotin-BSA-CBZ (96 s) could be alternatively conducted prior to detection. As it took 20 min to preincubate the CBZ sample with MA-CBZ, the indirect FO-BLI required 25 min in total to provide a result. Using a series of CBZ (0—0.1—0.5—1.0—2.0—3.0—4.0 µg/mL), a nonlinear dose–response curve was first generated in a buffer for detection (Figure 3B; R^2^ = 0.960, *n* =3). The LoD and the LLoQ were determined to be 0.05 and 0.1 µg/mL, respectively. The assay conditions in the biological media are summarized in Supplementary Table S1 (left column).

When applying the biosensor to biological media, the impact of complex components in the blood is a key issue to consider, as they adversely affect the sensor performance. The dilution of biological media was the first to be evaluated. A 5×, 10×, and 20× dilution using SD buffer resulted in similar performance for the two quality control samples at CBZ 0 and 0.5 µg/mL in healthy serum compared to that in pure SD buffer (Figure 3C). The 5× dilution factor was selected for continued optimization because of its potential to obtain a physiologically relevant detection range of 0.5 to 20 µg/mL. The use of high-BSA buffer (SD buffer containing 1% BSA) to dilute serum and the addition of Superblock buffer after surface immobilization failed to reduce nonspecific absorbance. Antibiofouling performance was achieved through a combination of high-salt SD buffer (SD buffer containing 270 mM of NaCl) with a 5× serum dilution. This was further ascribed to the negligible serum effect on the wavelength shifts of the two quality control samples. The same conditions were selected for whole-blood-based CBZ biosensing after a careful analysis. Nonlinear dose–response curves were generated after spiking CBZ from 0 to 4 µg/mL into the 5× serum (Figure 3B; R^2^ = 0.993, *n* = 3) and 5× whole blood (Figure 3B; R^2^ = 0.991, *n* = 3). Spiking one control sample at 6 µg/mL resulted in a back recovery of 108% and 91% in spiked serum and whole blood, respectively (*n* = 1). The indirect biosensor showed no cross-reactivity with valproate sodium, phenytoin sodium, and primidone in either matrix.

Among all the tested regeneration solutions, 500 mM phosphoric acid resulted in an overall recovery of 98–101% in shifts during the 12 regeneration cycles and showed negligible baseline drift over time with a total increase of 0.3 nm (Figure 3D; Figure S1). The results indicated the excellent stability and robustness of the indirect biosensor when used multiple times. Additionally, 10 mM glycine (pH 1.0) regenerated the sensors up to 12 times but caused a significant baseline drift of up to 2.2 nm (Supplementary Figure S1). A significant change in baseline shift was also observed with other solutions, in addition to their limited capability in regeneration.

### 3.3. Direct FO-BLI Biosensor for Detecting CBZ in Buffer, Serum and Whole Blood

**Figure 4:**
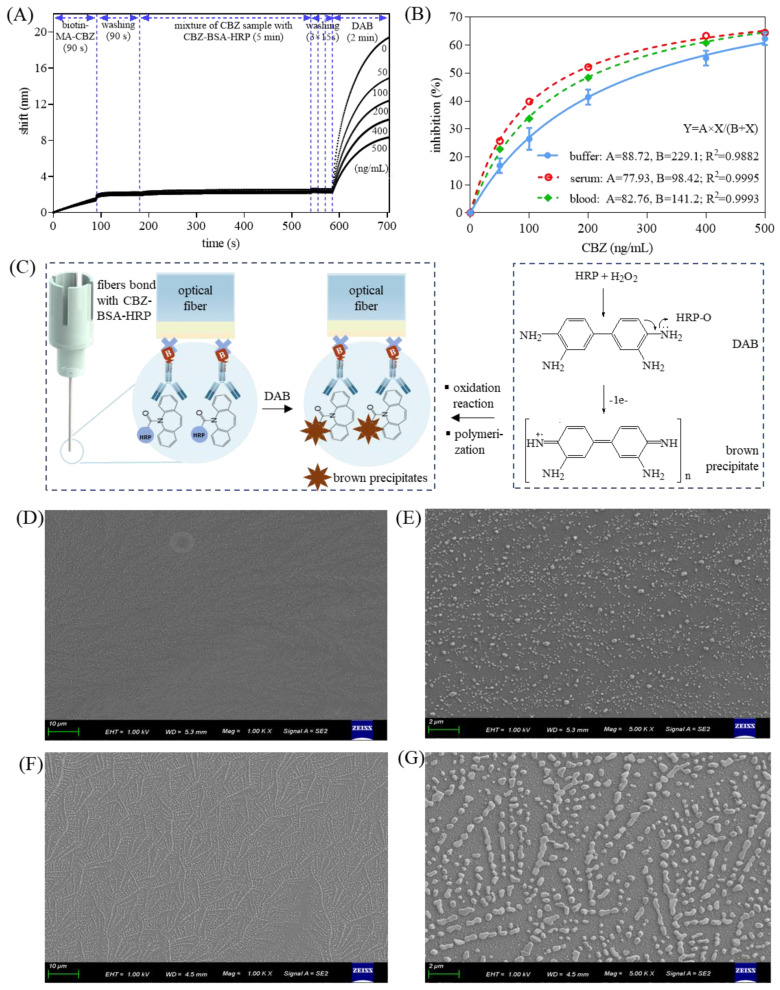
Direct FO-BLI biosensor for CBZ detection in buffer, serum, and whole blood: (**A**) Representative detection flowchart using DAB-induced signal amplification; (**B**) dose–response curves for CBZ detection in high-salt SD buffer, 20× diluted serum, and 20× whole blood. Error bars represent standard deviations (*n* = 3). (**C**) The oxidation reaction and polymerization to generate brown precipitate at the sites of HRP; (**D**–**G**) SEM images of the fiber tips when submerged into DAB solution to react with HRP bonded on CBZ-BSA to attain signals up to 5 nm (**D**,**E**) and 20 nm (**F**,**G**).

The fact that small-molecule drugs certainly result in limited wavelength shifts in a directly competitive binding interaction highlights the necessity of using a signal enhancer. Our recent work in quantifying SARS-CoV-2 antibodies and spike proteins revealed that the combination of HRP with the metal precipitate DAB was effective in amplifying the shifts and improving the detection. Thus, a combination of HRP conjugated CBZ-BSA with DAB was first used to improve the detection sensitivity for CBZ. Under a pre-optimized concentration of DAB at 200×, the CBZ-BSA-HRP concentration of 0.1 µg/mL was selected to obtain a signal threshold of 20 nm. The flowchart in Figure 4A reveals the entire detection process for the direct FO-BLI biosensor. Using the pre-functionalized sensors, the direct FO-BLI took approximately 7.5 min in total to deliver a result and required no sample preincubation. CBZ from 0 to 500 ng/mL in buffer generated a nonlinear dose–response curve (Figure 4B; R^2^ = 0.9882, *n* = 3). The LoD and LLoQ were determined to be 10 and 50 ng/mL, respectively.

Following the abovementioned steps to minimize the effect of serum and blood on sensor performance, the optimal conditions of 5× serum and 5× whole blood in high-salt SD buffer were selected (Figure S2A). Smooth nonlinear dose–response curves were produced for both the spiked serum and whole blood (Figure 4B). Although the detection profiles only showed an upper detection range of 500 ng/mL, corresponding to 10 µg/mL of CBZ in serum and whole blood, the upper limit could be tuned to reach of 16 µg/mL or higher for clinical use (Figure S2B). The assay conditions of the direct FO-BLI in the two biological media are summarized in Supporting Table S1 (right column). Spiking one control sample at 6 µg/mL resulted in a back recovery of 94 ± 15% (*n* = 2) and 105 ± 6% (*n* = 2) in spiked serum and whole blood, respectively. In addition, no cross-reactivity towards valproate sodium, phenytoin sodium, and primidone was observed at their maximum therapeutic concentrations in both matrices.

Later, the study evaluated the possibility of using gold nanoparticles and quantum dots to enhance signals by covalent bonding with CBZ-BSA (Figure S3). Only limited signals (less than 0.5 nm over 30 min) increased when associating the conjugates with preloaded MA-CBZ (data not shown). Data indicated that both nanomaterials may not be suitable for enhancing FO-BLI signals.

### 3.4. Characterization of DAB-Induced Precipitation on Optical Fiber Tips

In the direct FO-BLI, significant wavelength shifts were induced by the increased mass in the vicinity of the fiber tip surface. The increase mass was a result of the deposition of a water-insoluble brown precipitate at the site of HRP in the fiber tips, generated through the oxidization reaction of DAB with HRP bonded on CBZ-BSA (Figure 4C). Surface characterization of the fiber tips covered with DAB-induced principate was investigated by field emission scanning electron microscopy (SEM) (Zeiss Gemini500 SEM). When two fibers were submerged in the 200× DAB solution for 2 min, the optical fiber that produced a 5 nm wavelength shift (due to a small amount of residual CBZ-BSA-HRP) exhibited scattered precipitates on the surface (Figure 4D,E), whereas the optical fiber that generated a 20 nm shift (due to a high amount of residual CBZ-BSA-HRP) exhibited a clear, structured layer of precipitates with larger molecule sizes (Figure 4F,G). A more intensive, structured layer of precipitates with in-creased sizes is closely related to increased signal outputs. Such an intensive layer of precipitates on the tips could not be regenerated under all the tested regeneration solutions, indicating the one-time-use character of these active fibers (data not shown).

### 3.5. Validation of the Direct FO-BLI Biosensor with Paired Sera/Blood from CBZ-Treated Patients

The direct FO-BLI biosensor outperformed the indirect FO-BLI in multiple aspects, particularly with respect to its higher sensitivity, short turnaround time, and a more straightforward process without preincubation, increasing its usability in clinical diagnostics. Thus, the direct FO-BLI biosensor was further validated for its clinical utility in detecting CBZ samples from epileptic patients. Given that the final goal was to detect CBZ directly from whole blood, six paired sera and blood samples (referred to as S1 to S6) were obtained from CBZ-treated adult patients. Sera were diluted 5× and whole blood was diluted 20× using high-salt SD buffer before analysis. Each sample was measured four times using individual fibers in different runs. Independently, blood samples were pre-treated to determine CBZ using the UPLC technique.

**Figure 5:**
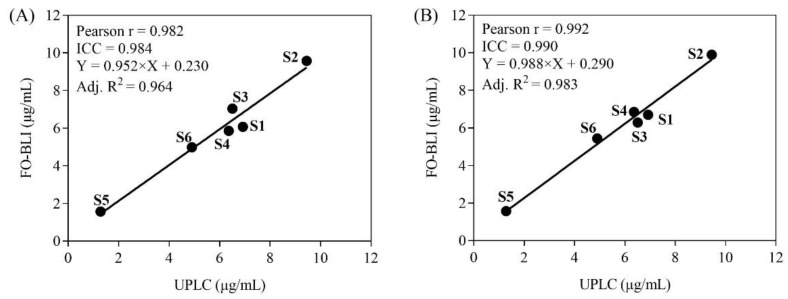
Evaluation of the direct FO-BLI biosensor with serum (**A**) and whole blood (**B**) samples from epileptic patients as compared with the conventional UPLC method.

The CBZ measurements obtained using both methods are summarized in Table 1. In direct FO-BLI, an overall inter-run variation of 7–14% was obtained for the six serum samples and 0–12% for the six whole blood samples. In UPLC, excellent repetition between the two runs was observed. Comparison of CBZ concentrations in sera obtained from the two different methods revealed a Pearson’s correlation coefficient of 0.982 (*p* = 0.0005; Figure 5A) with an ICC of 0.984. Comparison of CBZ concentrations in whole blood between the two methods revealed a Pearson r correlation coefficient of 0.992 (*p* = 0.0005; Figure 5B) and an ICC of 0.990. The difference in determining both sample matrices by the two techniques was illustrated visually using Bland–Altman plots, as shown in Figure S4. These revealed that the average differences were close to zero. Additionally, the whole blood/serum ratio as determined by the FO-BLI biosensor was 1.05 ± 0.10, with a range of 0.89 to 1.17. Compared to other CBZ bioassays, the direct FO-BLI biosensor showed multiple advantages, including the short time to result, full automation, being easy to operate, and importantly, reporting CBZ values directly from whole blood (Table 2).

## 4. Discussion and Conclusions

This study reports the development of two types of FO-BLI biosensors for on-site monitoring CBZ in serum and whole blood. The indirect FO-BLI biosensor, based on an indirectly competitive binding interaction, showed its capability to specifically detect CBZ within its clinically therapeutic range without interference from other anti-epileptic drugs. The indirect FO-BLI had an advantage in being regenerated up to 12 times with the use of 500 mM phosphoric acid, which reduced the test cost due to its capacity for multiple uses. However, the requirement to pre-mix each sample with the (commercial) target antibody on the one hand increased the cost and, on the other, resulted in a slow (25 min of assay time) and complex operation method. Given these limitations, the indirect FO-BLI was considered to be less effective when being implemented in modern clinical workflows. Conversely, the direct FO-BLI biosensor contributed to a much faster (approximately 7.5 min of time to result) and more straight-forward (by simple mixture) operation method. This was achieved by simply adding the sample into the CBZ-BSA-HRP conjugate of an equal volume for direct competitive binding towards the immobilized target antibody. Additionally, a much lower detection sensitivity of 10 ng/mL was obtained using a chromogen DAB to significantly simplify the signals without increasing significant background interreference. Unfortunately, the precipice-based signal enhancement failed to regenerate the sensors for the purpose of multiple uses. Despite this, the direct FO-BLI biosensor still maintains a reasonable cost per test as the biotinylated antibody and the HRP conjugated CBZ-BSA can be used for long term in addition to extra dilution before application. Additionally, a 20× final dilution of both the sera and blood into a high-salt SD buffer-minimized matrix interference to the biosensor performance. Further validation using six paired sera and whole blood samples from CBZ-treated epileptic patients demonstrated an excellent agreement between the direct FO-BLI and the benchmarked UPLC.

Altogether, the direct FO-BLI biosensor possesses multiple novel advantages, including the short turnaround time, full automation, and the delivery of CBZ values directly from whole blood, making it an effective and appropriate tool for on-site monitoring of CBZ in clinical practice. Additionally, the proposed technique can be applied for both single and high-throughout measurements. The limitation of the direct FO-BLI biosensor lies in the inability of the sensors to be regenerated for multiple uses in order to reduce costs. To address this, our next step is to investigate alternative effective signal enhancers for the direct FO-BLI. One bio-/nanomaterial that can both enhance signals (leading to a high sensitivity) and enable regeneration (reducing costs by reuse in multiple times) will greatly benefit the direct FO-BLI for clinical use.

## Figures and Tables

**Table 1:** Concentrations of the six paired sera and whole blood samples taken simultaneously from CBZ-treated epileptic patients and measured by both the direct FO-BLI biosensor and the UPLC. SD represents standard deviation of the mean and errors represent standard error of the mean.

Clinical Samples	Direct FO-BLI Biosensor (µg/mL, *n* = 4; in Serum)	Director FO-BLI Biosensor (µg/mL, *n* = 4; in Whole Blood)	UPLC (*n* = 2)
S1	6.07 ± 0.40	6.70 ± 0.65	6.92 ± 0.03
S2	9.57 ± 0.92	9.90 ± 0.82	9.45 ± 0.02
S3	7.04 ± 0.76	6.29 ± 0.77	6.51 ± 0.00
S4	5.86 ± 0.61	6.85 ± 0.15	6.36 ± 0.01
S5	1.57 ± 0.17	1.57 ± 0.18	1.28 ± 0.00
S6	4.98 ± 0.79	5.44 ± 0.02 *	4.90 ± 0.00

Note: * S6 in whole blood was only measured twice due to less enough volume.

**Table 2:** Comparison of the bioassays’ performance of the proposed FO-BLI biosensor with CBZ detection techniques reported in prior-art publications.

Technique	Principle	Assay Time	Sample	LoD	Clinical Utility	Operation	Ref.
LC-MS/MS	mass-to-charge ratios	10 min	dried blood	N/A	confirmed	half automated	[12]
PEDOT glassy carbon electrode	electrodes modified with MIP for direct binding	45 min	buffer	231 µg/mL	not studied	by hand	[13]
Immunochromatographic strip	CBZ-happen competes with sample to bind AuNPs modified MA-CBZ	10 min	serum	0.25–1.0 ng/mL	confirmed	by hand	[16]
Nanoplasmonic electrical field enhanced resonating	AuNPs modified MA-CBZ for direct binding	70 min	saliva, serum, whole blood	400 fg/mL	not studied	by hand	[17]
Suspension array fluorescence immunoassay	CBZ-happen coupled beads compete with sample to bind MA-CBZ	15 min	wastewater	0.14 ng/mL	only for wastewater samples	by hand	[18]
Direct FO-BLI biosensor	CBZ-BSA-HRP competes with sample to bind MA-CBZ	~7.5 min	serum, whole blood	10 ng/mL	confirmed	automated	this work

Note: AuNPs, gold nanoparticles; CBZ, carbamazepine; CBZ-BSA-HRP, CBZ-bovine serum albumin-horseradish peroxidase; FO-BLI, fiber-optic biolayer interferometry; LC-MS/MS, liquid chromatography mass spectrometry; LoD, limit of detection; MA-CBZ, monoclonal antibody towards CBZ; MIP, molecular imprinted polymer; N/A, not available; PEDOT, poly(3,4-ethylenedioxythiophene).

## Data Availability

Not applicable.

## Supplementary Materials

The following are available online at https://www.mdpi.com/article/10.3390/bios11120516/s1, Figure S1: The real-time displayed profiles of the indirect FO-BLI biosensor when regenerated up to 12 times using two different solutions, Figure S2: Controlling of non-specific binding from serum and a broader detection range of the direct FO-BLI biosensor, Figure S3: The covalent binding between AuNPs and QDs and CBZ-BSA, followed by the evaluation of AuNPs and QDs on signal enhancement in the direct FO-BLI biosensor, Figure S4: Bland–Altman comparison of CBZ measurements by the direct FO-BLI biosensor and the UPLC in the sera and whole blood, Table S1: Assay performance between the two FO-BLI biosensors for CBZ detection in sera and whole blood.

## References

[1] Thijs R.D., Surges R., O’Brien T.J., Sander J.W. (2019). Epilepsy in adults. Lancet.

[2] Ding D., Zhou D., Sander J.W., Wang W., Li S., Hong Z. (2021). Epilepsy in China: Major progress in the past two decades. Lancet Neurol..

[3] Singh G., Sander J.W. (2020). The global burden of epilepsy report: Implications for low- and middle-income countries. Epilepsy Behav..

[4] Duncan J.S., Sander J.W., Sisodiya S.M., Walker M.C. (2006). Adult epilepsy. Lancet.

[5] Santulli L., Coppola A., Balestrini S., Striano S. (2016). The challenges of treating epilepsy with 25 antiepileptic drugs. Pharmacol. Res..

[6] Arfman I.J., Wammes-van der Heijden E.A., Ter Horst P.G.J., Lambrechts D.A., Wegner I., Touw D.J. (2020). Therapeutic Drug Monitoring of Antiepileptic Drugs in Women with Epilepsy Before, During, and After Pregnancy. Clin. Pharmacokinet..

[7] Patsalos P.N., Spencer E.P., Berry D.J. (2018). Therapeutic Drug Monitoring of Antiepileptic Drugs in Epilepsy: A 2018 Update. Ther. Drug Monit..

[8] Greenberg R.G., Melloni C., Wu H., Gonzalez D., Ku L., Hill K.D., Hornik C.P., Zheng N., Jiang W., CohenWolkowiez M. (2016). Therapeutic index estimation of antiepileptic drugs: A systematic literature review approach. Clin. Neuropharmacol..

[9] Sommerfeld-Klatta K., Zielińska-Psuja B., Karaźniewcz-Łada M., Główka F.K. (2020). New Methods Used in Pharmacokinetics and Therapeutic Monitoring of the First and Newer Generations of Antiepileptic Drugs (AEDs). Molecules.

[10] Hammoud A., Nguyen D.K., Sawan M. (2018). Detection Methods and Tools of Administered Anti-Epileptic Drugs—A Review. Bio-sens. Bioelectron. Open Acc..

[11] Zabihollahpoor A., Rahimnejad M., Najafpour-Darzi G., Moghadamnia A.A. (2020). Recent advances in electroanalytical methods for the therapeutic monitoring of antiepileptic drugs: A comprehensive review. J. Pharm. Biomed. Anal..

[12] D’Urso A., Rudge J., Patsalos P.N., de Grazia U. (2019). Volumetric Absorptive Microsampling: A New Sampling Tool for Thera-peutic Drug Monitoring of Antiepileptic Drugs. Ther. Drug Monit..

[13] Hammoud A., Chhin D., Nguyen D.K., Sawan M. (2021). A new molecular imprinted PEDOT glassy carbon electrode for carbam-azepine detection. Biosens. Bioelectron..

[14] Bian S., Zhu B., Rong G., Sawan M. (2021). Towards wearable and implantable continuous drug monitoring: A review. J. Pharm. Anal..

[15] Ates H.C., Roberts J.A., Lipman J., Cass A.E.G., Urban G.A., Dincer C. (2020). On-Site Therapeutic Drug Monitoring. Trends Biotechnol..

[16] Zhou S., Xu L., Liu L., Kuang H., Xu C. (2020). Development of a monoclonal antibody-based immunochromfatographic assay for the detection of carbamazepine and carbamazepine-10, 11-epoxide. J. Chromatogr. B..

[17] Inci F., Filippini C., Baday M., Ozen M.O., Calamak S., Durmus N.G., Wang S., Hanhauser E., Hobbs K.S., Juillard F. (2015). Multitarget, quantitative nanoplasmonic electrical field-enhanced resonating device (NE2RD) for diagnos-tics. Proc. Natl. Acad. Sci. USA.

[18] Carl P., Sarma D., Gregório B.J.R., Hoffmann K., Lehmann A., Rurack K., Schneider R.J. (2019). Wash-Free Multiplexed Mix-and-Read Suspension Array Fluorescence Immunoassay for Anthropogenic Markers in Wastewater. Anal. Chem..

[19] Garzón V., Pinacho D.G., Bustos R.-H., Garzón G., Bustamante S. (2019). Optical Biosensors for Therapeutic Drug Monitoring. Biosensors.

[20] Ong J.J., Pollard T.D., Goyanes A., Gaisford S., Elbadawi M., Basit A.W. (2021). Optical biosensors—Illuminating the path to per-sonalized drug dosing. Biosens. Bioelectron..

[21] Overacker R.D., Plitzko B., Loesgen S. (2021). Biolayer interferometry provides a robust method for detecting DNA binding small molecules in microbial extracts. Anal. Bioanal. Chem..

[22] Gao S.X., Zheng X., Wu J.H. (2017). A biolayer interferometry-based competitive biosensor for rapid and sensitive detection of saxitoxin. Sens. Actuators B Chem..

[23] Petersen R.L. (2017). Strategies Using Bio-Layer Interferometry Biosensor technology for vaccine research and development. Biosensors.

[24] Bian S., Shang M., Sawan M. (2021). Rapid biosensing SARS-CoV-2 antibodies in vaccinated healthy donors. Biosens. Bioelectron..

[25] Masson J.F. (2020). Consideration of Sample Matrix Effects and “Biological” Noise in Optimizing the Limit of Detection of Biosensors. ACS Sens..

[26] Xu J., Lee H. (2020). Anti-Biofouling Strategies for Long-Term Continuous Use of Implantable Biosensors. Chemosensors.

