# Supplementary material for: On-Site Biolayer Interferometry-Based Biosensing of Carbamazepine in Whole Blood of Epileptic Patients
Source: Biosensors (Basel). 2021 Dec 15;11(12):516. doi: 10.3390/bios11120516 (PMC8699405; doi:10.3390/bios11120516)
Supplement: Supplementary file 1 [file biosensors-11-00516-s001.zip › biosensors-1470661-supplementary.pdf]

Supplementary Materials

MATERIALS AND METHODS

1.1 Ultra-Performance Liquid Chromatography (UPLC)

Chromatographic analysis was conducted using an ACQUITY UPLC H-class system coupled with a PDA detector (Waters, USA). To prepare a UPLC standard curve, a stock solution of CBZ at 100 μg/mL was prepared by dissolving CBZ (1.0 mg) in methanol (10 mL) via ultrasound, followed by serial dilutions to obtain final CBZ concentrations at 0.2, 1, 2, 4, 6, and 8 μg/mL by spiking the serum. For the pretreatment of patient blood, all samples were centrifuged at 4000 rpm for 10 min to separate plasma from blood cells, followed by dilution in methanol to obtain the same 5× matrix condition. Both the standards and plasma were vortex-mixed for 1 min and centrifuged at 9000 rpm for 10 min to remove proteins. The supernatants were filtered using 0.22 μm syringe filters and degassed prior to analysis. Chromatographic separation of CBZ was conducted at 35 °C on a reversed-phase ACQUITY UPLC BEH C18 column (C18, 50 × 2.1 mm; 1.7 μm particle size). A gradient elution was applied at a flow rate of 0.4 mL/min with a mobile phase composed of water and methanol. A wavelength of 285 nm was selected for detection. Each sample was measured twice.

1.2 Bioconjugation of CBZ-BSA with nanomaterials for enhancing signals

Conjugation of CBZ-BSA with gold nanoparticles (AuNPs; diameter of 20 nm, 20 OD) was conducted through covalent bonding between carboxylic group from surface of AuNPs and ε-amine groups from lysine residues of BSA, following the protocol of Gold Conjugation Kit (20 nm, 20 OD) ((ab188215); Abcam, Shanghai, China). The CBZ-BSA-AuNPs conjugates were centrifuged at 9000 *g* for 20 min to remove the unbound and obtained a stock concentration at 5 OD prior to use. Conjugation of CBZ-BSA with quantum dots (QDs; Wuhan Jiayuan Quantum Dots Co., Ltd., Wuhan, China) was conducted through covalent bonding as well between carboxylic functionalized QDs (QDs-COOH) and lysine residues of BSA. Briefly, 100 μL of 2 μM QDs-COOH in PBS was gently mixed with 300 μL of 2 mg/mL CBZ-BSA in PBS (a molar ratio of 45 versus QDs-COOH). The interaction was initiated when adding 25 μL of 10 mg/mL of N-(3-Dimethylaminopropyl)-N-ethylcarbodiimide hydrochloride (EDC) in MES buffer (a molar ratio of 4000 versus QDs-COOH) into the mixture and remained 2 h under gentle shaking at 25 °C. Afterwards, the solution was centrifugated at 8000 rpm for 3 min to remove any precipitates. The collected supernatants were further purified five times using

10,000 MWCO PES-typed ultrafiltration devices (Sartorius Stedim Biotech GmbH, Gottingen, Germany) under 25 °C. The synthesized bioconjugates were stored in 4 °C prior to use.

RESULTS

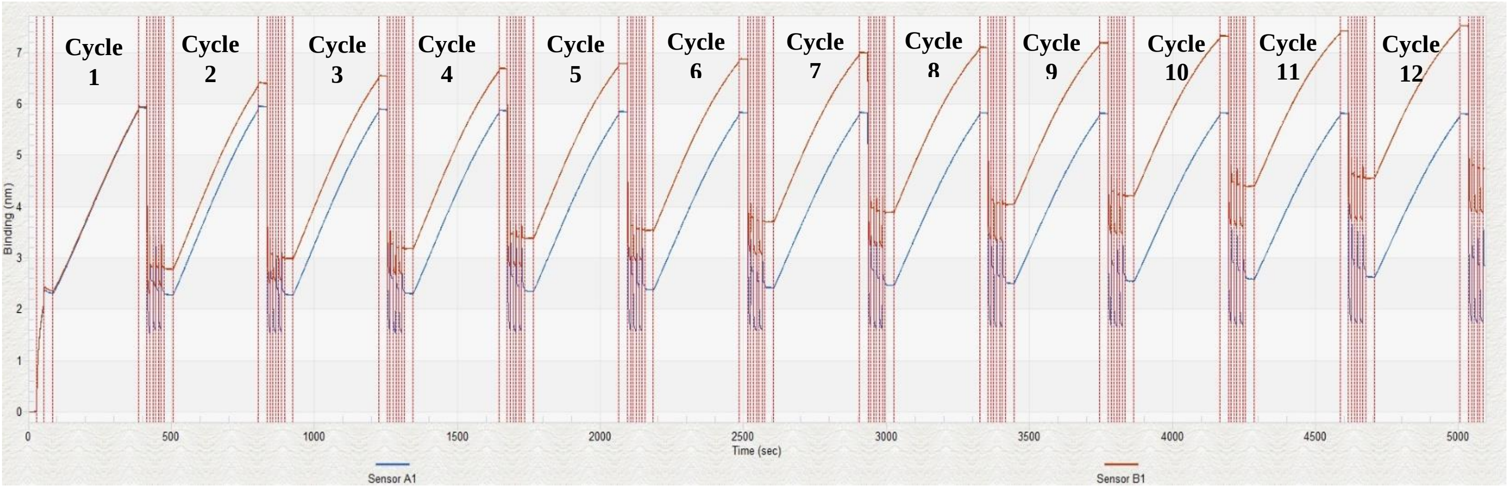

Figure S1. The real-time displayed profiles of the indirect FO-BLI biosensor when regenerated up to 12 times using two different solutions. Sensor A1, in blue, represents the profile when using 500 mM phosphoric acid as the regeneration solution. Sensor B1, in red, represents the profile when using 10 mM Glycine pH 0.5 as the regenerartion buffer. The 500 mM phosphoric acid-induced regeneartion resulted in a negligible baseline shift over time with a total of 0.32 nm of baseline shift increase. The 10 mM Glycine pH 0.5-based regeneration caused strong baseline shift up to 2.20 nm of shift increase. FO-BLI, fiber optic biolayer interferometry.

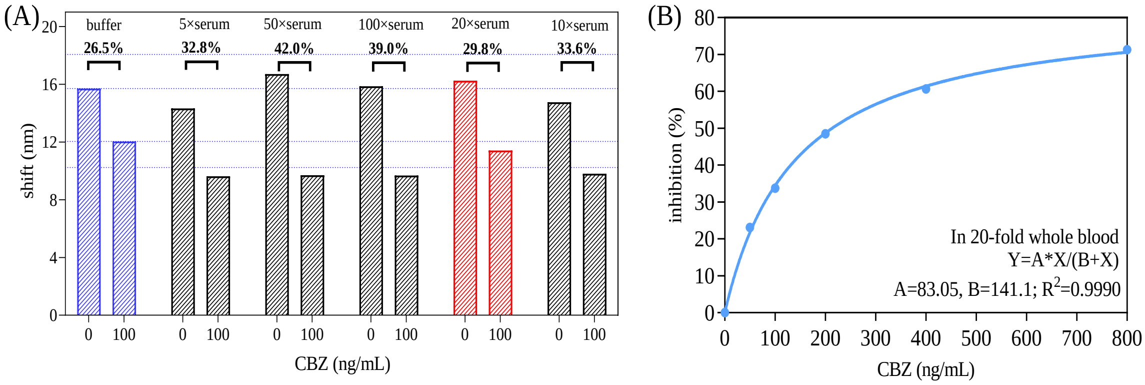

Figure S2. Controlling of non-specific binding from serum and a broader detection range of the direct FO-BLI biosensor. (A) The shifts of CBZ at 0 and 100 ng/mL under series dilution of healthy

control serum into the high-salt SD buffer as compared to the shifts obtained with pure SD buffer. In particular, the two upper dashed lines represent the shift of initial CBZ at 0 ng/mL in pure SD buffer and its 115% signal criteria line; the two bottom dashed lines represent the shift of initial CBZ of 100 ng/mL in pure SD buffer and its 85% signal criteria line. (B) Non-linear dose–concentartion curve for detecting CBZ in a broader range up to 800 ng/mL, corresponding to 16 μg/mL in whole blood, which is sufficient to detect CBZ within its therapeutic reference range. CBZ, carbamazepine; SD, sample diluent.

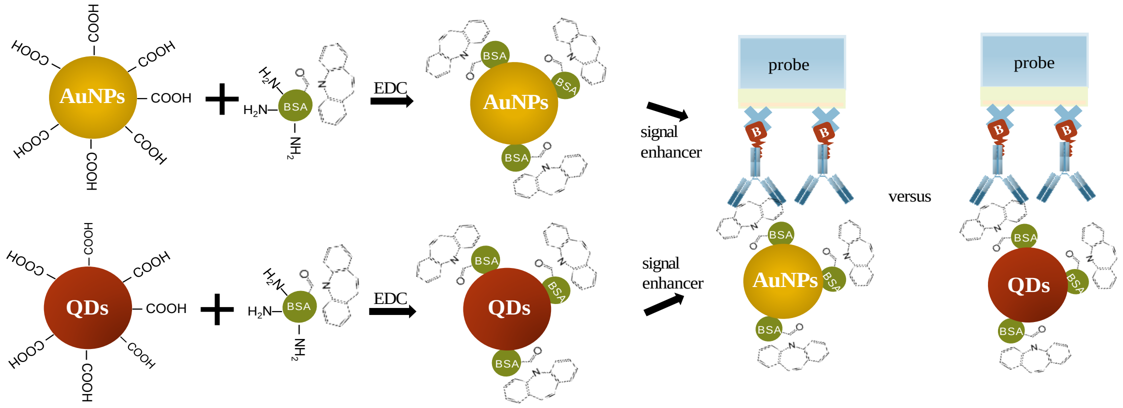

Figure S3. The covalent binding between AuNPs and QDs and CBZ-BSA, followed by the evaluation of AuNPs and QDs on signal enhancement in the direct FO-BLI biosensor. AuNPS, gold nanoparticles; CBZ-BSA, CBZ-bovine serum albumin conjugate, QDs, quantum dots.

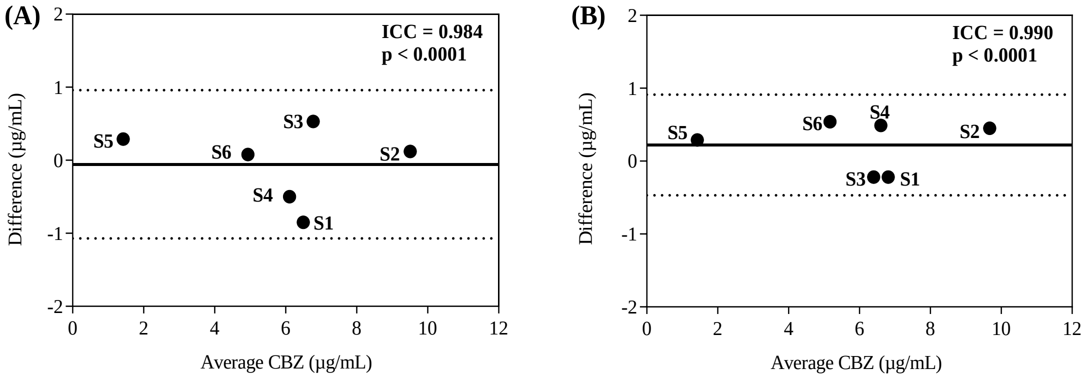

Figure S4. Bland–Altman comparison of CBZ measurements by the direct FO-BLI biosensor and the UPLC in the sera (A) and whole blood (B). The absolute difference and the average between the two measurements (μg/mL) is plotted in the Y-axis and the X-axis, respectively.

Dark lines mean the average of the difference and dotted lines mean the 95% limits of agreement. UPLC, Ultra-Performance Liquid Chromatography.

Supplementary Table S1. Assay performance between the two FO-BLI biosensors for CBZ detection in sera and whole blood.

**Main steps**	**Items**	**The indirect FO-BLI biosensor**	**The direct FO-BLI biosensor**
Probe Immobilization	Principle	indirect competitive binding	direct competitive binding
	Probes	SA sensor	SA sensor
	**Capture molecule**	Biotin-BSA-CBZ	Biotin-MA-CBZ
	Capture shift	1.77 ± 0.08 nm (n = 18)	1.60 ± 0.08 nm (n = 20)
Competitive Binding of Sample	Sample matrix	serum, whole blood	serum, whole blood
	Sample dilution	5 ×	20 ×
	Competitive binding agent	MA-CBZ	CBZ-BSA-HRP
	Competitive binding agent conc.	10 μg/mL	0.1 μg/mL
	Pre-incubation time	20 min	N/A
	Detection time	5 min	5 min
Signal Enhancement	**Enhancer**	N/A	DAB
	Enhancer conc.	N/A	200 ×
	Enhancing time	N/A	2 min
Assay Properties	Detection range	0.1 - 4 μg/mL	10 – 500 ng/mL
	LOD	50 ng/mL	10 ng/mL
	LLOQ	100 ng/mL	50 ng/mL
	Sample volume	20 μL	5 μL
	Sample-to-result time	25 min	~7.5 min
Comparison	Detection range	Within physiologically relevant concentrations range	Improved sensitivity for lower CBZ concentration detection
	Sample pretreament	Need of sample pre-incubation	No need of sample pre-incubation
	Toxicity of materials	No use of any less environment-friendly materials	Potential toxicity of DAB
	**Costs**	Use the MA-CBZ per sample per test (~4000 rmb for 1 mL of 1 mg/mL of MA-CBZ); sensors can be regenerated up to 12 times	Biotinlated MA-CBZ can be used for long period; Sensors are one-time-use only (approximately 40 rmb per one fiber)

Notes: CBZ, carbamazepine; CBZ-BSA-HRP, CBZ-bovine serum albumin-horseradish peroxidase; DAB, 3,3'-diaminobenzidine; FO-BLI, fiber optic biolayer interferometry; LoD, limit of detection; LLoQ, lower limit of quantification; MA-CBZ, monoclonal antibody against carbamazepine; N/A, not applicable; SA sensors, streptavidin sensors.
